# Albumin as a Prognostic Marker for Atrial Fibrillation Recurrence following Cryoballoon Ablation of Pulmonary Venous

**DOI:** 10.3390/jcm12010264

**Published:** 2022-12-29

**Authors:** Nili Schamroth Pravda, Gregory Golovchiner, Gustavo Goldenberg, Ygal Plakht, Maya Wiessman, Shir Tal, Alon Barsheshet, Ehud Kadmon, Aharon Erez, Keren Skalsky, Tzlil Grinberg, Inbar Nardi Agmon, Yaron Aviv, Ran Kornowski, Arthur Shiyovich, Ashraf Hamdan

**Affiliations:** 1 Department of Cardiology, Rabin Medical Center, Petach Tikva 4941492, Israel; 2 Affiliated to Faculty of Medicine, Tel Aviv University, Tel Aviv 69978, Israel; 3 Department of Nursing, Faculty of Health Sciences, Ben-Gurion University of the Negev, Beer-Sheva 84105, Israel

**Keywords:** atrial fibrillation, pulmonary vein isolation, recurrence, albumin

## Abstract

Introduction: Atrial fibrillation (AF) recurrence following pulmonary vein isolation (PVI) ablation has clinical significance. Identifying risk factors for AF recurrence is important. We investigated serum albumin (SA) levels (g/dL) as a prognostic factor for the recurrence of AF following cryoballoon PVI ablation. Methods: We included patients who underwent cryoballoon PVI ablation at our institution between the years 2013 and 2018. The primary outcome was recurrence of AF during follow up. Results: Our cohort consisted of 126 patients (67% males, mean age 61.8 ± 10.0 years). The pattern of AF amongst the cohort was paroxysmal in 62.5%, persistent in 25.4%, and longstanding persistent in 6.3%. Those with lower SA levels had a mean AF duration significantly less than those with higher SA levels (2.81 years, 7.34 years, and 6.37 years for SA levels of <3.8, 3.8–4.1, and ≥4.1, respectively; *p* = 0.003). Patients with lower SA levels were significantly more likely to have had more previous cardioversions and a larger left atrial area and volume. The mean follow-up was 380 days, in which the AF recurrence rate was 20.6%. Patients with lower SA level had significantly more AF recurrences (47.4%, 16.7%, and 2.2% for SA levels of <3.8, 3.8–4.1, and ≥4.1, respectively; *p* < 0.001). Upon multivariate analysis, an SA level < 3.8 was associated with a higher risk of AF recurrence (OR = 5.422 95% CI 1.134; 25.910; *p* < 0.001). Conclusion: SA levels were found to be a strong independent marker for AF recurrence following PVI ablation.

## 1. Introduction

Atrial fibrillation (AF) is the most common sustained arrhythmia worldwide, with a prevalence of up to 4% of the adult population [1]. It is associated with significant morbidity and mortality [2]. The maintenance of sinus rhythm is associated with improved symptom control and improvement of quality of life [2]. There is increasing evidence that this strategy is also associated with improved cardiovascular outcomes [3]. AF catheter ablation with pulmonary vein isolation has proven to be an effective and safe alternative to anti-arrhythmic drugs [4,5]. However, AF recurrence rates following pulmonary vein isolation (PVI) ablation are substantial. In contemporary randomized control trials, recurrence rates range up to 34–43% at one year following the procedure [6,7]. Various risk factor for AF recurrence have been described, and these should be taken into consideration in shared decision making with the patient [2]. Studies have shown that albumin levels are associated with the incidence of new onset AF [8,9]. Albumin as a prognostic marker of AF recurrence following PVI ablation has yet to be investigated. We investigated serum albumin (SA, g/dL) levels as a prognostic factor for the recurrence of AF following PVI ablation.

## 2. Materials and Methods

We included 126 consecutive patients with AF who had cryoballoon PVI ablation performed at our institution between the years 2013 and 2018. Patients with an inflammatory/rheumatic condition, active infection, acute illness, active malignancy, major surgery in the prior 6 months, and chronic liver disease/cirrhosis, and patients with a previous PVI ablation were excluded from this study (*n* = 9). Retrospectively, patient demographic data, echocardiographic parameters, and procedural data were collected from patient files. Medical conditions were obtained from patient clinic files and according to the International Classification of Diseases, Ninth Revision, Clinical Modification (ICD-9-CM) codes, if available. Patients with AF were classified according to the Heart Rhythm Society expert consensus statement as follows: paroxysmal, persistent, and longstanding persistent [8,9]. Each patient file was reviewed, and the data of AF duration were taken from hospital records according to the dates recorded of initial diagnosis of atrial fibrillation on the electronic filing system. Symptoms alone without documentation of atrial fibrillation were not regarded as sufficient.

### 2.1. Ablation

The ablation procedure was performed under conscious sedation with midazolam and fentanyl or under general anesthesia. All PVI ablation procedures included in this cohort underwent cryoballoon PVI ablation. The PVI ablation was performed with a 15 F steerable trans-septal sheath (FlexCath), an Arctic Front Advance cryoballoon catheter, and an Achieve Mapping Catheter (Medtronic, Minneapolis, MN, USA). This procedure was done under fluoroscopic guidance and after trans-septal puncture, as previously described [10]. In brief, the cryoballoon was inserted into the pulmonary vein antrum and was then filled with a refrigerant to cool and occlude the tissue with the aim of creating electrical isolation of the tissue, as seen on electrophysiological recordings. The cooling was stopped if the temperature dropped below −55 degrees Celsius [11].

The procedure endpoint was electrical isolation between the pulmonary veins and left atrium, as confirmed with electrical entrance block. Patients were followed in our outpatient clinic.

### 2.2. Study Groups

Valid ambulatory SA levels within 24 h prior to the PVI ablation were obtained. This was included as part of the routine laboratory tests done prior to the procedure. SA levels were classified (as tertiles) as follows: T1, <3.8; T2, 3.8 to 4.19; and T3, ≥4.2 g/dL. SA levels were measured using a photometric color test for clinical chemistry analyzers.

### 2.3. Follow Up and Outcomes

Follow up duration was 1 year. Follow up was performed at 3, 6, 9, and 12 months. Prior to each follow up visit, patients underwent ambulatory Holter monitoring for 48 h. Antiarrhythmic drugs were ceased at the discretion of the treating physician at the 3-month follow up visit. Anticoagulation treatment was continued in all patients for at least 3 months, and then it was continued or ceased according to the stroke risk based on the CHA_2_DS_2_VASc score. Documentation of arrhythmia was taken from clinic notes and patient file documentation. The primary outcome was AF recurrence during follow up, which was defined as at least one episode of atrial arrhythmia that lasted more than 30s on Holter monitoring or symptomatic AF documented on electrocardiogram in patients’ files after a 3-month blanking period (therapy stabilization period) post ablation. If patients were symptomatic and complained of palpitations, they were invited for additional clinical review and Holter monitoring.

The study was a retrospective analysis and was conducted in compliance with the principles outlined in the declaration of Helsinki, and it was approved by the institutional Ethics Committee.

### 2.4. Statistical Analysis

Statistical analysis was performed using IBM SPSS 26 (SPSS Inc., Chicago, IL, USA) software. Patient characteristics were presented as mean ± standard deviation (SD) for continuous variables; range, median, and interquartile range (IQR) for count data; and numbers (n) and percent (%) for categorical data. Comparisons of baseline and follow up characteristics between the study groups were performed using Chi-square tests for categorical variables, non-parametric Kruskal–Wallis tests for count data, and analysis of variance (ANOVA) tests for continuous variables.

Associations between SA level (in the presented categories) and the risk recurrent AF in the univariate and multivariate levels were assessed using binomial logistic regression. The primary independent variable (SA) was used in the multivariable model using the Enter method. Other investigated independent variables were introduced to the model using the forward stepwise method. The results of the models were presented as the regression coefficients (B) and their standard errors (SE), odds ratios (OR)/adjusted odds ratios (adjOR), and 95% confidence intervals (CIs) for OR. For each test, *p* < 0.05 was considered statistically significant.

## 3. Results

Our cohort consisted of 126 patients, of whom 67% were male with mean age of 61.8 ± 10.0 years. Baseline characteristics of the total cohort and according to the SA tertiles are shown in Table 1, Table 2 and Table 3. Prominent comorbidities amongst the cohort included hypertension (59%), dyslipidemia (46.8%), and coronary artery disease (22.2%). The majority of the cohort had good left ventricular function (62.5%). The pattern of AF amongst the cohort was mostly paroxysmal in 62.5%, persistent in 25.4%, and long standing persistent in 6.3%. A quarter of the cohort had previous atrial flutter, and 20.6% had previously undergone cavotricuspid isthmus ablation for atrial flutter. The majority of the cohort (92.9%) had previously been treated with an antiarrhythmic drug, of which the most commonly used were propafenone (51.6%) followed by amiodarone (50.0%).

### 3.1. Comparison across SA Tertiles

Those with lower SA levels had mean AF durations significantly shorter than those with higher SA levels (2.81 years, 7.34 years, and 6.37 years in those with SA levels of T1, T2, and T3, respectively; *p* = 0.003). Patients with lower SA levels were significantly more likely to have had more previous cardioversions than those with higher SA levels (*p* = 0.002). Notably, left atrial areas were significantly larger with lower SA tertiles (24.97 ± 4.84 cm^2^, 22.79 ± 5.09 cm^2^, and 21.55 ± 4.46 cm^2^ in T1, T2, and T3, respectively; *p* = 0.024). There were also significant differences in mean left atrial volumes (mL/m^2^) in accordance with SA level (136.24 ± 42.56, 119.76 ± 34.96, and 105.54 ± 27.73 in T1, T2, and T3, respectively; *p* = 0.002).

### 3.2. Follow up and Outcome

**Figure 1:**
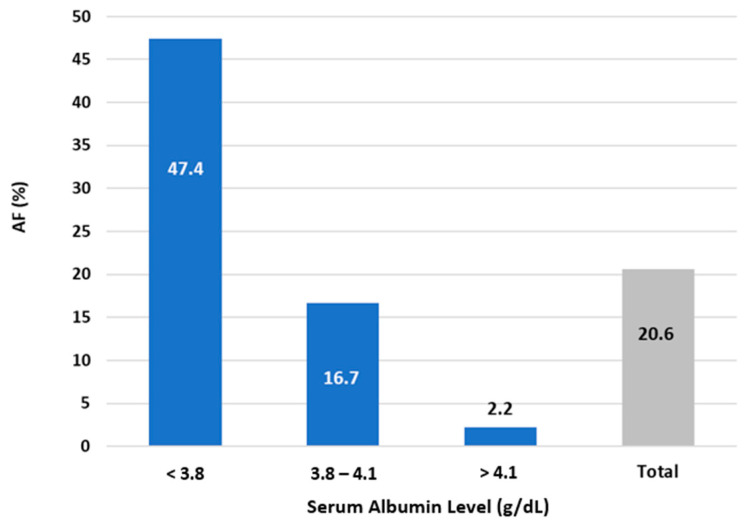
Atrial fibrillation (AF) recurrence rate following pulmonary vein isolation (PVI) ablation according to serum albumin (SA) level group.

Follow up data are shown in Table 4. There were follow up data for 123 participants, of which the mean follow up was 380 days. The overall AF recurrence rate was 20.6% (n = 26), as shown in Figure 1. Patients in the lower SA tertile had significantly more AF recurrences (47.4%, 16.7%, and 2.2% in T1, T2, and T3, respectively; *p* < 0.001). During the 3-month blanking period following the PVI ablation, those with higher SA had significantly fewer recurrences compared to those with lower SA levels (24.34%, 12.2%, and 4.8%, in T1, T2, and T3, respectively; *p* = 0.037).

### 3.3. Association between SA Level and Recurrent AF

On univariate analysis SA levels < 3.8g/dL were nine times more likely to have a recurrence of AF following PVI ablation [OR = 9.00 (95% CI 3.424; 23.653), *p* < 0.001], compared to those with albumin levels ≥ 3.8g/dL. The results of the multivariate model (Table 5) showed a statistically significant association between SA and AF recurrence, with the lower categories of SA independently associated with a higher rate of AF recurrence following PVI ablation. Three parameters were found to be strong independent markers for AF recurrence upon multivariate analysis: SA levels > 3.8 with a adjOR = 5.422 (95% CI 1.134; 25.910), *p* < 0.001, followed by left atrial diameter with an adjOR = 1.206 (95% CI 1.026; 1.416), *p* = 0.023, for every 1 cm increase in diameter as well as direct current cardioversion with an adjOR = 2.596 (95% CI 1.411; 4.777), *p* = 0.002. The pattern of AF (paroxysmal vs. persistent/long persistent) was a significant marker for AF recurrence on univariate analysis with OR = 2.849 (95% CI 1.170; 6.944), *p* = 0.021, but this was not a significant factor on multivariable analysis.

## 4. Discussion

Our study’s main finding is that albumin is a strong independent prognostic marker of AF recurrence following PVI ablation. Upon multivariate analysis, those with SA levels lower than 3.8g/dL were 5.4 times more likely to have AF recurrence following PVI ablation compared to patients with SA ≥ 3.8 g/dL. This was more influential than the risk associated with left atrial diameter or previous cardioversion. To our knowledge, this is the first study that has investigated the prognostic value of SA as a risk factor for AF recurrence.

Epidemiological data are growing, linking low SA levels to the incidence of a variety of cardiovascular diseases [12]. Recent data are accumulating linking albumin as a risk factor in the incidence of AF [13,14]. On an epidemiological level, albumin was shown by Liao et al. to be inversely associated to the future risk of AF in a population-based prospective cohort without pre-existing AF (hazard ratio (HR) = 0.90 (95% CI 0.86; 0.94) [14]. Van Beek et al. [13] showed a similar association amongst patients in intensive care. Lower SA levels were associated with the early development of AF during admission as well as with an increased number of AF episodes. This association was linear, with a lowered risk of AF by 14% (95% CI 3%; 23%) for every gram per deciliter increase in SA. We focused our study on albumin as a biomarker that has been researched in the cardiovascular sphere and is known to have many important biochemical properties. We did not use total serum protein for risk stratification, as this measure includes many proteins, including albumin and globulins. The globulin fraction is made up of different proteins, including immunoglobulins, enzymes, and carrier proteins, which all have important physiological roles, including those in the immune system. We focused our risk stratification on albumin in an aim to be more specific and to not be confounded by these other protein fractions.

The postulation is that albumin is a marker of the nutritional status of the patients and is an indicator of underlying health status. However, there are further physiological reasons that albumin could be an important disease risk factor. Firstly, the physiological properties of SA are multiple and are related to inflammatory properties, the coagulation pathway, and anti-oxidant activity [12,15,16]. Albumin is a negative acute phase protein, and hypoalbuminemia is a maker of a heightened inflammatory state of the patient. Indeed, several inflammatory biomarkers have been shown to be associated with the incidence and perpetuation of AF. Both c-reactive protein and interleukin-6 have been associated with AF recurrence following catheter ablation [17,18]. It is hypothesized that cardiac fibroblasts are stimulated by inflammation mediators, which contribute to atrial fibrosis and the ensuing atriopathy, which increases the risk and persistence of AF [19].

We found that patients with lower SA levels had significantly shorter durations of AF. We hypothesize that this could be due to lower SA levels being a marker of the chronicity of comorbid condition systemic diseases, and AF may be a later manifestation of various comorbid conditions such as advancing age, hypertension, etc. [2].

Albumin also binds both endogenous and exogenous substances. It is an important factor in drug bioavailability affecting drug pharmacokinetics, duration of drug activity, and metabolism of drugs [20]. It also has a fundamental role in maintaining plasma colloid osmotic pressure, preventing extravascular fluid extravasation [21]. Furthermore, SA levels have been associated with increased extracellular volume, a noninvasive marker of myocardial fibrosis on magnetic resolution imaging, in patients with various cardiovascular diseases such as aortic stenosis and heart failure [19,22]. Lower albumin could be a marker of myocardial fibrosis and could be a marker of the atrial fibrosis in patients with AF. Indeed, we found that lower SA levels were significantly associated with more previous cardioversions for AF as well as larger atrial areas and volumes. This could be a surrogate marker for the severity of the atriopathy of AF and thus could be associated with an increased risk of recurrence following PVI ablation.

Upon multivariate analysis, we found that AF recurrence following PVI ablation was significant, with a cut-off of SA levels lower than 3.8g/dL. SA levels less than 3.5g/dL are usually regarded as the cut-off for hypoalbuminemia. However, it has been shown that albumin levels within the lower normal range of 3.5–4.0g/dL have an association with poorer outcomes in other cardiovascular conditions [23]. A decreased admission serum albumin level has been found to be an independent predictor of long-term mortality in hospital survivors of acute myocardial infarction [24]. It is important to note that patients with low SA levels, as well as lower normal range SA, are at increased risk of AF recurrence following PVI ablation.

Our study is the first to show a significant association between SA levels and recurrence of AF following PVI ablation. Recurrence of AF has clinical significance, not only in terms of patients’ symptomatology, but also in terms of cardiovascular outcomes. The EAST-AFNET 4 Trial found that early rhythm control was significantly associated with a lower risk of adverse cardiovascular outcomes. This trial was ended prematurely due to a significantly lower composite of death from cardiovascular causes, stroke, or hospitalization with worsening of heart failure or acute coronary syndrome amongst those with early rhythm control [3]. PVI ablation has been shown to be an effective form of rhythm control. However, contemporary randomized control trials have shown that the documentation of AF recurrence following the procedure remains significant [6,10]. Identification of risk factors can assist in selecting patients who will have a higher chance of maintaining sinus rhythm following the procedure.

Multiple risk factors for AF recurrence have been identified, with many being linked to atrial size and function, atriopathy associated with AF, and the nature of AF [25,26,27]. Our study is the first to suggest that albumin could be a modifiable risk factor for AF recurrence.

Our study has limitations that include the retrospective design of the study and a relatively small sample size, which also limited our ability to add additional AF risk factors into the modelling of outcomes. It is important to note that our study found an association between SA and AF recurrence following PVI ablation. However, our study was not a mechanistic study, and our findings are hypothesis generating and not mechanistic in nature. Renal function was not assessed as correlated to AF recurrence following PVI ablation, and this is a further limitation. Endocardial mapping was not done in our cohort, and the relationship between quantity of low voltage areas and SA levels was not assessed. The outcome was collected from 24–48-h Holter monitoring done during 3-month periods or documentation in patient file data. In many cases it was not possible to report the exact date of recurrence. Accordingly, a logistic regression model was used and not a Cox regression model.

## 5. Conclusions

In our single-center cohort, SA levels were found to be a strong independent marker for AF recurrence following PVI ablation. This relationship may be multifactorial in nature, and further research is needed to clarify this association.

## Figures and Tables

**Table 1:** Baseline characteristics (demographics and comorbidities) according to serum albumin (SA) level group.

**Variable**	**Values**	**Serum Albumin Level (g/dL)**				***p* Value**
		**<3.8**	**3.8–4.1**	**≥4.1**	**Total**	
	*n*	38	42	46	126	
Demographics						
Sex	Male	21/38 (55.3%)	29/42 (69.0%)	34/44 (77.3%)	84/124 (67.7%)	0.102
Age, years	*n*	38	42	44	124	
	Mean (SD)	60.92 (9.44)	63.1 (8.90)	61.43 (11.59)	61.84 (10.05)	0.597
Anthropometrics						
Weight, kg	*n*	38	42	46	126	
	Mean (SD)	84.83 (17.81)	84.88 (14.24)	86.78 (13.81)	85.56 (15.16)	0.793
Height, cm	*n*	38	42	46	126	
	Mean (SD)	169.55 (9.21)	173.55 (8.15)	170.98 (7.40)	171.4 (8.32)	0.091
BMI, kg/m^2^	*n*	38	42	46	126	
	Mean (SD)	29.25 (5.58)	27.94 (3.34)	29.37 (4.97)	28.86 (4.71)	0.304
Comorbidities						
Hypertension		25/38 (65.8%)	23/42 (54.8%)	24/42 (57.1%)	72/122 (59%)	0.578
Diabetes mellitus		12/38 (31.6%)	9/42 (21.4%)	10/46 (21.7%)	31/126 (24.6%)	0.490
Dyslipidemia		18/38 (47.4%)	19/42 (45.2%)	22/46 (47.8%)	59/126 (46.8%)	0.968
Smoker		4/38 (10.5%)	4/41 (9.8%)	5/45 (11.1%)	13/124 (10.5%)	0.979
Coronary artery disease		9/38 (23.7%)	9/42 (21.4%)	10/46 (21.7%)	28/126 (22.2%)	0.966

BMI = body mass index, SD = standard deviation.

**Table 2:** Atrial fibrillation (AF) characteristics (at the baseline) according to serum albumin (SA) level group.

**Variable**	**Values**	**Serum Albumin Level (g/dL)**				***p* Value**
		**<3.8**	**3.8–4.1**	**≥4.1**	**Total**	
	*n*	38	42	46	126	
AF characteristics						
AF duration, years	*n*	34	34	35	103	
	Mean (SD)	2.81 (1.99)	7.34 (6.76)	6.37 (6.72)	5.52 (5.91)	0.003
Type of AF	Paroxysmal	29/38 (76.3%)	28/42 (66.7%)	30/46 (65.2%)	87/126 (69.0%)	0.196
	Persistent	5/38 (15.8%)	11/42 (26.2%)	15/46 (32.6%)	32/126 (25.4%)	
	Longstanding persistent	4/38 (10.5%)	3/42 (7.1%)	1/46 (2.2%)	8/126 (6.3%)	
Number of previous cardioversions	Range	0–8	0–5	0–4	0–8	
	Median (IQR)	0 (0–2)	1 (0–1)	0 (0–0)	0 (0–1)	0.022
	0	19/35 (54.3%)	16/35 (45.7%)	28/35 (80%)	63/105 (60%)	0.002
	1	4/35 (11.4%)	13/35 (37.1%)	3/35 (8.6%)	20/105 (19.0%)	
	≥2	12/35 (34.3%)	6/35 (17.1%)	4/35 (11.4%)	22/105 (21.0%)	
NYHA	Range	0–2	0–3	0–3	0–3	
	Median (IQR)	1 (0–1)	1 (0–1)	1 (0.5–1)	1 (0–1)	0.576
	0	12/34 (35.3%)	9/35 (25.7%)	9/37 (24.3%)	30/106 (28.3%)	0.501
	1	18/34 (52.9%)	21/35 (60.0%)	23/37 (62.2%)	62/106 (58.5%)	
	2	4/34 (11.8%)	4/35 (11.4%)	2/37 (5.4%)	10/106 (9.4%)	
	3	0/34 (0%)	1/35 (2.9%)	3/37 (8.1%)	4/106 (3.8%)	
Canadian Cardiovascular Society (CCS) Severity of Atrial Fibrillation (SAF) scale	Range	1–4	1–4	0–4	0–4	
	Median (IQR)	3 (2–3)	3 (2–3)	2 (1–3)	3 (2–3)	0.061
	0	0/34 (0%)	0/39 (0%)	1/37 (2.2%)	1/110 (0.8%)	0.433
	1	2/34 (5.3%)	6/39 (14.3%)	9/37 (19.6%)	17/110 (13.5%)	
	2	11/34 (28.9%)	13/39 (31%)	12/37 (26.1%)	36/110 (28.6%)	
	3	18/34 (47.4%)	18/39 (42.9%)	14/37 (30.4%)	50/110 (39.7%)	
	4	3/34 (7.9%)	2/39 (4.8%)	1/37 (2.2%)	6/110 (4.8%)	
Previous atrial flutter		7/38 (18.4%)	8/42 (19.0%)	17/46 (37.0%)	32/126 (25.4%)	0.078
Previous CTI ablations		8/38 (21.1%)	6/42 (14.3%)	12/46 (26.1%)	26/126 (20.6%)	0.392
CHADS score	Range	0–4	0–5	0–4	0–5	
	Median (IQR)	1.5 (0–2)	1 (0–2)	1 (0 to –2)	1 (0–2)	0.458
	0	10/38 (26.3%)	16/42 (38.1%)	14/45 (31.1%)	40/125 (32.0%)	0.758
	1	9/38 (23.7%)	11/42 (26.2%)	14/45 (31.1%)	34/125 (27.2%)	
	2	14/38 (36.8%)	10/42 (23.8%)	13/45 (28.9%)	37/125 (29.6%)	
	3	4/38 (10.5%)	2/42 (4.8%)	3/45 (6.7%)	9/125 (7.2%)	
	≥4	1/38 (2.6%)	3/42 (7.1%)	1/45 (2.2%)	5/125 (4.0%)	
CHA₂DS₂-VASc Score	Range	0–6	0–7	0–5	0–7	
	Median (IQR)	2 (0.8–3.3)	2 (0–3)	2 (0.5–3)	2 (0–3)	0.965
	0	9/38 (23.7%)	14/42 (33.3%)	11/45 (24.4%)	34/125 (27.2%)	0.524
	1	7/38 (18.4%)	4/42 (9.5%)	10/45 (22.2%)	21/125 (16.8%)	
	2	9/38 (23.7%)	7/42 (16.7%)	6/45 (13.3%)	22/125 (17.6%)	
	3	4/38 (10.5%)	10/42 (23.8%)	10/45 (22.2%)	24/125 (19.2%)	
	4	6/38 (15.8%)	2/42 (4.8%)	5/45 (11.1%)	13/125 (10.4%)	
	5	2/38 (5.3%)	3/42 (7.1%)	3/45 (6.7%)	8/125 (6.4%)	
	≥6	1/38 (2.6%)	2/42 (4.8%)	0/45 (0%)	3/125 (2.4%)	

AF = atrial fibrillation, IQR = interquartile range, NYHA = New York Heart Association, CTI = cavotricuspid isthmus, SD = standard deviation.

**Table 3:** Treatment and echocardiogram measurements (at the baseline) according to serum albumin (SA) level group.

**Variable**	**Values**	**Serum Albumin Level (g/dL)**				***p* Value**
		**<3.8**	**3.8–4.1**	**≥4.1**	**Total**	
	*n*	38	42	46	126	
Treatment						
Number of antiarrhythmic drugs	Range	0–5	0–4	0–5	0–5	
	Median (IQR)	1 (1–2)	1 (1–2)	1 (1–3)	1 (1–2)	0.414
	0	6/38 (15.8%)	1/42 (2.4%)	2/46 (4.3%)	9/126 (7.1%)	0.171
	1	17/38 (44.7%)	23/42 (54.8%)	23/46 (50.0%)	63/126 (50.0%)	
	≥2	15/38 (39.5%)	18/42 (42.9%)	21/46 (45.7%)	54/126 (42.9%)	
Amiodarone		18/38 (47.4%)	22/42 (52.4%)	23/46 (50.0%)	63/126 (50.0%)	0.905
Propafenone		17/38 (44.7%)	22/42 (52.4%)	26/46 (56.5%)	65/126 (51.6%)	0.556
Flecanide		9/38 (23.7%)	12/42 (28.6%)	19/46 (41.3%)	40/126 (31.7%)	0.194
Dronaderone		5/38 (13.2%)	4/42 (9.5%)	7/46 (15.2%)	16/126 (12.7%)	0.722
Sotalol		11/38 (28.9%)	10/42 (23.8%)	7/46 (15.2%)	28/126 (22.2%)	0.307
Aspirin		9/38 (23.7%)	11/42 (26.2%)	11/46 (23.9%)	31/126 (24.6%)	0.958
Warfarin		11/38 (28.9%)	8/42 (19%)	15/46 (32.6%)	34/126 (27.0%)	0.340
Dabigatran		4/38 (10.5%)	9/42 (21.4%)	3/46 (6.5%)	16/126 (12.7%)	0.099
Apixaban		10/38 (26.3%)	8/42 (19.0%)	11/46 (23.9%)	29/126 (23.0%)	0.731
Xarelto		11/38 (28.9%)	14/42 (33.3%)	9/46 (19.6%)	34/126 (27.0%)	0.330
ACE inhibitors		19/38 (50.0%)	10/42 (23.8%)	17/46 (37%)	46/126 (36.5%)	0.052
ARBs		6/38 (15.8%)	9/42 (21.4%)	3/46 (6.5%)	18/126 (14.3%)	0.130
Beta blockers		30/38 (78.9%)	30/42 (71.4%)	39/46 (84.8%)	99/126 (78.6%)	0.312
Calcium Channel Blockers		5/38 (13.2%)	7/42 (16.7%)	5/46 (10.9%)	17/126 (13.5%)	0.727
Digoxin		2/38 (5.3%)	1/42 (2.4%)	4/46 (8.7%)	7/126 (5.6%)	0.432
Echocardiogram measurements						
Left ventricular function	Good (>55%)	24/35 (68.6%)	21/35 (60.0%)	20/34 (58.8%)	65/104 (62.5%)	0.922
	Preserved (50–55%)	7/35 (20.0%)	11/35 (31.4%)	10/34 (29.4%)	28/104 (26.9%)	
	Mild (41–49%)	1/35 (2.9%)	0/35 (0%)	1/34 (2.9%)	2/104 (1.9%)	
	Moderate (30–40%)	2/35 (5.7%)	1/35 (2.9%)	2/34 (5.9%)	5/104 (4.8%)	
	Severe (<30%)	1/35 (2.9%)	2/35 (5.7%)	1/34 (2.9%)	4/104 (3.8%)	
Left atrial diameter (mm)	*n*	29	30	28	87	
	Mean (SD)	43.9 (5.79)	40.6 (6.79)	41.64 (3.66)	42.03 (5.71)	0.076
Left atrial area (cm^2^)	*n*	29	28	31	88	
	Mean (SD)	24.97 (4.84)	22.79 (5.09)	21.55 (4.46)	23.07 (4.95)	0.024
Systolic pulmonary artery pressure (mmHg)	*n*	24	22	24	70	
	Mean (SD)	27.96 (8.53)	25.77 (10.05)	28.13 (7.59)	27.33 (8.68)	0.602
Left atrial volume (mL/m^2^)	*n*	33	34	35	102	
	Mean (SD)	136.24 (42.56)	119.76 (34.96)	105.54 (27.73)	120.22 (37.29)	0.002

AF = atrial fibrillation, ARBs = angiotensin receptor blockers, IQR = interquartile range, SD = standard deviation.

**Table 4:** Follow up characteristics by serum albumin (SA) level group.

**Variable**	**Values**	**Serum Albumin Level (g/dL)**				***p* Value**
		**<3.8**	**3.8–4.1**	**≥4.1**	**Total**	
	*n*	38	42	46	126	
AF (during a 3-month window)		9/37 (24.3%)	5/41 (12.2%)	2/42 (4.8%)	16/120 (13.3%)	0.037
Canadian Cardiovascular Society (CCS) Severity of Atrial Fibrillation (SAF) scale	Range	0–3	0–3	0–2	0–3	
	Median (IQR)	0 (0–1)	0 (0–1)	0 (0–1)	1 (0–1)	0.897
	0	21/34 (61.8%)	21/40 (52.5%)	21/41 (51.2%)	63/115 (54.8%)	0.317
	1	8/34 (23.5%)	16/40 (40%)	19/41 (46.3%)	43/115 (37.4%)	
	2	4/34 (11.8%)	2/40 (5.0%)	1/41 (2.4%)	7/115 (6.1%)	
	3	1/34 (2.9%)	1/40 (2.5%)	0/41 (0%)	2/115 (1.7%)	
	4	0/34 (0%)	0/40 (0%)	0/41 (0%)	0/115 (0%)	
Number of hospitalizations	Range	0–6	0–2	0–7	0–7	
	Median (IQR)	0 (0–1)	0 (0–0)	0 (0–0)	0 (0–0)	0.103
	≥1	10/29 (34.5%)	5/38 (13.2%)	8/39 (20.5%)	23/106 (21.7%)	0.108
NYHA	Range	0–3	0–2	0–3	0–3	
	Median (IQR)	1 (0–1)	1 (0–1)	1 (0–1)	1 (0–1)	0.625
	0	15/35 (42.9%)	12/40 (30.0%)	16/42 (38.1%)	43/117 (36.8%)	0.465
	1	18/35 (51.4%)	26/40 (65.0%)	23/42 (54.8%)	67/117 (57.3%)	
	2	0/35 (0%)	2/40 (5.0%)	2/42 (4.8%)	4/117 (3.4%)	
	3	2/35 (5.7%)	0/40 (0%)	1/42 (2.4%)	3/117 (2.6%)	
Treatment						
Antiarrhythmic drug use		17/30 (56.7%)	19/35 (54.3%)	22/37 (59.5%)	58/102 (56.9%)	0.906
Amiodarone		8/38 (21.1%)	6/42 (14.3%)	8/46 (17.4%)	22/126 (17.5%)	0.728
Propafenone/flecainide		6/38 (15.8%)	5/42 (11.9%)	9/46 (19.6%)	20/126 (15.9%)	0.617
Dronaderone		0/38 (0%)	0/42 (0%)	1/46 (2.2%)	1/126 (0.8%)	0.416
Sotalol		2/38 (5.3%)	2/42 (4.8%)	1/46 (2.2%)	5/126 (4.0%)	0.732
Hydroquinidine		1/37 (2.7%)	0/42 (0%)	0/46 (0%)	1/125 (0.8%)	0.302

AF = atrial fibrillation, NYHA = New York Heart Association.

**Table 5:** Risk factors for atrial fibrillation (AF) recurrence following pulmonary vein isolation (PVI) ablation—univariate and multivariate analyses.

Parameter	Value	Univariate				Multivariate			
		B (SE)	OR	(95% CI)	*p*	B (SE)	AdjOR	(95% CI)	*p*
Serum albumin level (g/dL)	<3.8 vs. ≥3.8	2.197 (0.493)	9.000	(3.424; 23.653)	<0.001	1.960 (0.877)	7.101	(1.274; 39.575)	0.025
Left atrial diameter (mm)	1 mm increase	0.173 (0.053)	1.189	(1.071; 1.320)	0.001	0.198 (0.081)	1.219	(1.041; 1.429)	0.014
Previous cardioversion	Yes vs. no	0.678 (0.187)	1.971	(1.366; 2.843)	<0.001	0.774 (0.336)	2.168	(1.121; 4.191)	0.021
Type of baseline AF	Persistent/long persistent vs. paroxysmal	1.046 (0.454)	2.849	(1.170; 6.944)	0.021	1.181 (0.965)	3.257	(0.492; 21.739)	0.221

AF = atrial fibrillation, B = regression coefficient, SE = standard error, AdjOR = adjusted odds ratio, CI = confidence interval.

## Data Availability

The data presented in this study are available on request from the corresponding author. The data are not publicly available due to ethical restrictions and patient data that was taken from Rabin Medical Center.
